# Enhanced Vibrational Spectroscopies as Tools for Small Molecule Biosensing

**DOI:** 10.3390/s150921239

**Published:** 2015-08-28

**Authors:** Souhir Boujday, Marc Lamy de la Chapelle, Johannes Srajer, Wolfgang Knoll

**Affiliations:** 1 UPMC Univ Paris 6, UMR CNRS 7197, Laboratoire de Réactivité de Surface, 4 Place Jussieu, F-75005 Paris, France; E-Mail: souhir.boujday@upmc.fr; 2 CNRS, UMR 7197, Laboratoire de Réactivité de Surface, F-75005 Paris, France; 3 Center for Biomimetic Sensor Science, 50 Nanyang Drive, Singapore 637553, Singapore; 4 Université Paris 13, Sorbonne Paris Cité, Laboratoire CSPBAT, CNRS, (UMR 7244), 74 rue Marcel Cachin, F-93017 Bobigny, France; E-Mail: marc.lamydelachapelle@univ-paris13.fr; 5 AIT Austrian Institute of Technology, Donau City Strasse 1, A-1220 Vienna, Austria; E-Mail: johannes.srajer@ait.ac.at

**Keywords:** biosensors, infrared, plasmonic, enhanced spectroscopies, SERS, IRRAS

## Abstract

In this short summary we summarize some of the latest developments in vibrational spectroscopic tools applied for the sensing of (small) molecules and biomolecules in a label-free mode of operation. We first introduce various concepts for the enhancement of InfraRed spectroscopic techniques, including the principles of Attenuated Total Reflection InfraRed (ATR-IR), (phase-modulated) InfraRed Reflection Absorption Spectroscopy (IRRAS/PM-IRRAS), and Surface Enhanced Infrared Reflection Absorption Spectroscopy (SEIRAS). Particular attention is put on the use of novel nanostructured substrates that allow for the excitation of propagating and localized surface plasmon modes aimed at operating additional enhancement mechanisms. This is then be complemented by the description of the latest development in Surface- and Tip-Enhanced Raman Spectroscopies, again with an emphasis on the detection of small molecules or bioanalytes.

## 1. Introduction

Rapid and accurate detection of undesirable and toxic substances in the human environment has become a major concern in our modern society. Consequently, considerable effort is being devoted to the development of analytical devices enabling the detection of a target molecule, specifically and rapidly, even in trace amounts and even in demanding milieus. Biosensors allow the qualitative monitoring and even more the quantitative evaluation of the concentration of small analytes in a cocktail of (excess) molecules, small and large. A number of techniques, well-established for larger targets, e.g., proteins or DNA amplicons from PCR protocols, have been demonstrated to work in the lab; with some of them later even being successfully implemented in commercial products. Surface-plasmon resonance (SPR) based instruments are certainly the most prominent examples for this [1]. The sensitivities of these techniques could be further significantly enhanced by the introduction of labelling strategies, most prominently demonstrated in the ELISA approach [2] or by the various fluorescence array technologies that are on the market [3].

Accurate detection of small analytes is still an analytical challenge. Major difficulties become apparent whenever one is dealing with a diagnostic task for which either the analyte cannot be labeled or is simply too small for the attachment of a (chromophore) label. Examples would be micro RNA in body fluid samples [4], odorants [5] or volatile organic compounds (VOCs) as cancer markers in breath analysis [6]. For these cases other spectroscopic methods might offer solutions that do not need any labels attached to the analyte of interest and are even competitive in terms of sensitivity and selectivity.

In this short review we summarize some of the promising approaches based on recent development in various formats of infrared (IR) spectroscopy and in surface- and tip-enhanced Raman spectroscopies (SERS and TERS).

## 2. IR Spectroscopy Applied to Direct and Label-Free Sensing of Small Molecules

Surface IR spectroscopy was widely used for biosensors, first, to monitor the preparation of biosensors starting by the very preliminary surface functionalization step, often the formation of self-assembled monolayers (SAMs), and second, to investigate the attachment of biomolecules or receptors to the surfaces, and finally, as a transduction technique for biorecognition and binding events.

Various IR spectroscopy modes of operation are used for biosensors, including the simplest transmission mode. Yet, for the sensitive detection of adsorbed small molecules, the use of surface-enhancement mechanisms is mandatory. Among these modes, the most commonly used are: Attenuated Total Reflection InfraRed (ATR-IR) Spectroscopy, InfraRed Reflection Absorption Spectroscopy (IRRAS), eventually using the modulation of the polarisation (PM-IRRAS), and Surface Enhanced Infrared Spectroscopy (SEIRS). Hereafter, the principles of these modes are briefly described. Then, through examples, the input of these techniques in biosensors preparation will be discussed. In a third part, the efficiency of surface IR techniques for direct biosensing of small molecules will be exemplified. Finally, the last part of this section is devoted to localized surface-plasmon field-enhanced IR spectroscopy.

### 2.1. Surface-Enhanced IR Techniques

**Figure 1:**
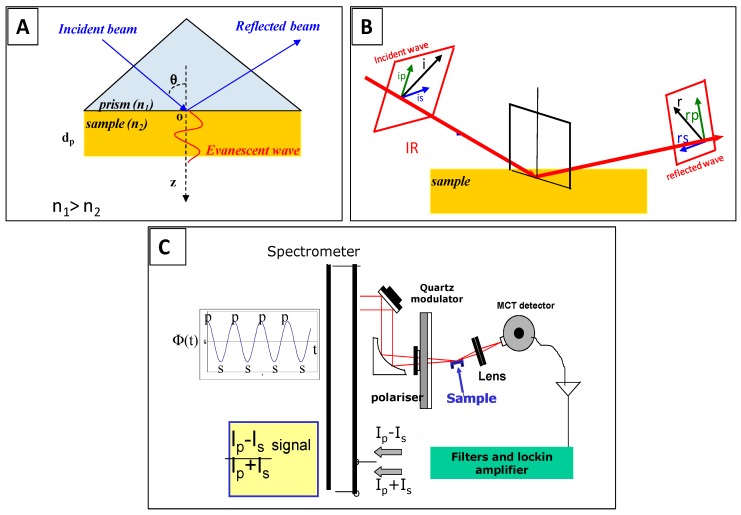
Surface IR spectroscopy techniques: (**A**) principle of Attenuated Total Reflection InfraRed (ATR-IR); (**B**) principle of (phase-modulated) InfraRed Reflection Absorption Spectroscopy (IRRAS/PM-IRRAS); and (**C**) experimental setup for PM-IRRAS measurements.

The attenuated total reflection (ATR) configuration utilizes the total internal reflection of the IR beam within a high refractive index material, at a certain angle of incidence above the critical angle for total internal reflection (Figure 1A). In this format, the sample is first pressed against the ATR crystal then probed by the evanescent wave, with a sensitivity decaying with the distance (Z) from the crystal interface [7,8,9].

For the InfraRed Reflection Absorption Spectroscopy (IRRAS, also called RAIRS), the incident IR beam is focused on the surface of the sample at grazing incidence and is subsequently reflected by the substrate. The standing wave generated near the surface is maximal for p-polarisation at this incidence and leads to the enhancement of the corresponding electric field component normal to the surface, Ep, and consequently, to an increase of the signal of the molecules perpendicular to the surface (Figure 1B,C) [10,11]. This enhancement allows modulating the polarization of the incident beam between p and s bring an additional benefit as the contributions of isotropic environment are not recorded and spectra are collected with a real-time normalization; no reference measurement is needed. 

Finally, Surface Enhanced Infrared Spectroscopy (SEIRS) like Surface Enhanced Raman Scattering (SERS, *cf.* below), benefits from a plasmonic enhancement effect on nanostructured (noble metal) surfaces, although the sensitivity increase is often less spectacular than for SERS enhancement [12]. SEIRS measurements are often done in the ATR configuration [13].

### 2.2. Surface IR Techniques to Monitor Biosensor Preparation

Perfect reliability and reproducibility are needed for biosensors and this can only be achieved through a molecular-level control of each preparation step. The control of biosensing surface preparation is very challenging, mainly due to the complexity of the successive elementary steps, *i.e.*, surface pre-treatment, functionalization and SAM formation, biomolecules immobilization, *etc*. The input of Surface IR in monitoring all these steps is significant as very few techniques allow for the characterization of this substrate/adsorbate interface. Surface IR was efficiently used to monitor all these steps. Spectroscopic data are mainly collected in two wavenumber regions: the region from 2800 to ~3000 cm^−1^ for SAMs characterization (CH_2_ stretching vibrations, *cf.* below) and the region between 1400 and 2000 cm^−1^ for biomolecules survey (amide, amine, acid and phosphate vibrations) [14].

**Figure 2:**
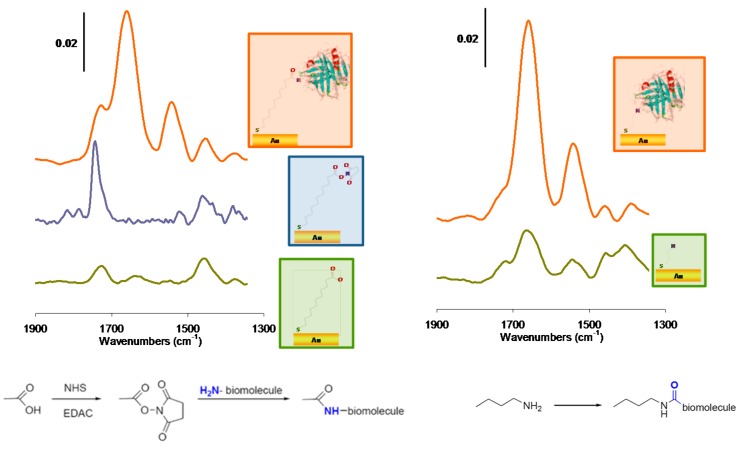
Step by step monitoring of β-lactoglobulin immobilization on acid-terminated SAMs (**left**, mercaptoundecanoic acid grafting, activation, then protein immobilization and on amine-terminated SAMs (**right**, cysteamine grafting and attachment of activated protein), adapted from reference [24].

IRRAS and PM-IRRAS mode were widely used to characterize the grafting and assembly of functional thiols on gold to form self-assembled monolayers allowing subsequent grafting of biomolecules [15,16,17,18,19,20,21]. Firstly, information on the successful grafting are sought and obtained through the presence of the characteristic vibration modes of functional groups. Figure 2 illustrates a typical monitoring upon gold surfaces modification by acid (here mercaptoundecanoic acid) and amine (cysteamine in this case)-terminated thiols. The formation of the acid-terminated SAMs, starting from mercaptoundecanoic acid is evidenced on gold surfaces by the appearance of IR absorption bands of the symmetric *ν*_COO−_ and the δ_CH2_ around 1450 cm^−1^ and also the *ν*_COOH_ at 1723 cm^−1^. The subsequent activation of the acid functions using NHS-EDC can also be verified by IR. On the spectra, this is evidenced by the decrease of carbonyl vibration band of the carboxylic acids, and by the presence of new carbonyl stretching vibrations: O=C−O−NR group at 1741 cm^−1^, and the carbonyl stretching vibrations of the succinimide at 1817 and 1787 cm^−1^, respectively. Finally, protein grafting leads to amide bands I and II at *ν =* 1650 cm^−1^ and *ν =* 1550 cm^−1^, respectively [22,23,24]. On amine-terminated SAMs, the deformation symmetric vibration *δ*_NH_ of primary ammonium functions are present at 1575 cm^−1^ together with the stretching vibration *δ*_NH_ of primary amine functions and the deformation asymmetric vibration *δ*NH of primary ammonium functions at 1660–1630 cm^−1^. In this case also protein grafting is evidenced by the amide bands. It is important to note that the collected data also provides quantitative information on the adsorbed layer; peak area integration can be compared and correlated to the surface coverages [25]. The decomposition of the amide bands also provides valuable information on the secondary and tertiary structure of proteins.

**Figure 3:**
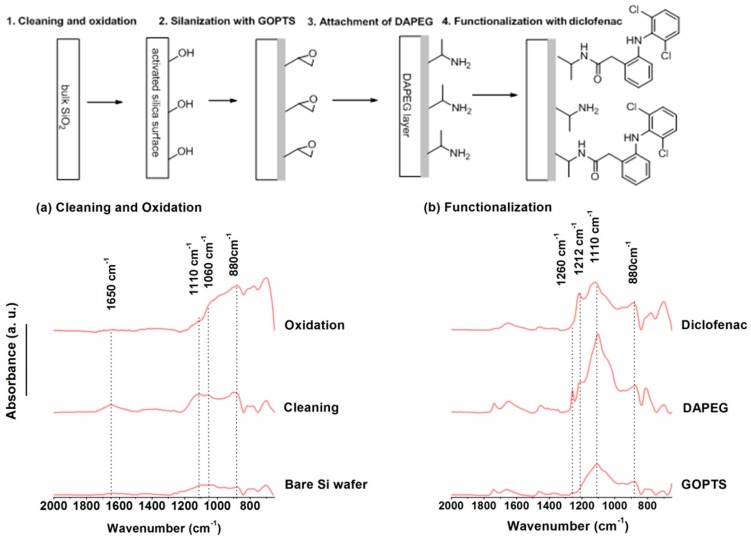
Preparation of a PEG based diclofenac biosensor chip or microarray followed at each step of cleaning and functionalization by Surface IR in Attenuated Total Reflection (ATR) mode, adapted from reference [32].

For oxide surface analysis ATR is often preferred [26,27,28,29] though the use of IRRAS or PM-IRRAS was reported [30,31]. Oxide surface functionalization by silanes requires, besides the classical cleaning, an oxidation step aiming at generating additional OH terminal groups. Figure 3 exemplifies the elementary characterization of the successive steps required to design a biosensor for haptens on a silica-glass micro-arrays [32]. The target molecule in this example is a small pharmaceutical molecule, diclofenac, the detection of which requires robust surfaces, stable against regeneration and specific to the desired adsorption. FT-IR allows for following the cleaning through the removal of pollutants (absence of CH_2_ stretching vibrations) but also the oxidation step through the increase of Si-O-Si stretching around 1110 cm^−1^ together with the intensification of the Si-OH band in the 3600 cm^−1^ region. The subsequent functionalization by epoxy functions and poly(ethylene glycol) (PEG) to avoid non-specific adsorption are also observable by IR spectroscopy through the bands in the 1100–1200 cm^−1^ region. Finally, the latest step of preparation of this biosensor, namely, the immobilization of the target molecule on the surface in order to proceed to a competitive detection, is also observed by ATR.

### 2.3. Surface IR Techniques for Small Molecules Biosensing

The input of surface IR in the quantification of surface functionalization is significant. Nevertheless, Surface IR is also a valuable technique to monitor biorecognition events. Its usefulness for protein and bacteria detection was demonstrated [33], but the results in small molecule sensing, which detection by label-free techniques is often challenging, are more spectacular. The particular case of DNA binding and hybridization was widely studied [34,35,36]. Both grafting and hybridization can be monitored by IR. ssDNA grafting leads to a very characteristic spectrum of the DNA phosphodiester backbone (broad band at 1188 cm^−1^), and the asymmetric PO_2_^−^ stretching mode at 1238 cm ^−1^ and many additional vibrations due to the bases in the 1400–1700 cm^−1^ region. The hybridization is evidenced by the appearance of the band at ca 1605 cm^−1^, attributed to changes in hydrogen bonding between complementary bases.

The use of peptide nucleic acids, PNAs makes the IR detection of hybridization events even easier [37,38]. PNA fragment grafting on gold surfaces leads to intense bands at 1549–1270 cm^−1^; while upon hybridization of complementary DNA the presence of the characteristic IR signature of phosphate chemical groups, P-O stretch vibration, in PO_2_^−^, at *n* = 1237 cm^−1^ and *n* = 1081 cm^−1^, respectively, reveals the formation of PNA-DNA heteroduplex and allows for the label-free detection of nucleic acid targets in biological solutions [37,38].

Surface IR can be successfully utilized to detect the presence of some small targets provided that they have specific IR signatures. This is the case for polyaromatic hydrocarbons (PAHs), a class of urban pollutants resulting from the incomplete combustion of organic substances such as fossil fuels [39]. These compounds are identified carcinogens and display also endocrine-disruptive activity. The five-ring derivative benzo[a]pyrene (B[a]P), whose concentration often correlates well with the total PAHs contents in environmental samples, is monitored as marker of PAHs. 

**Figure 4:**
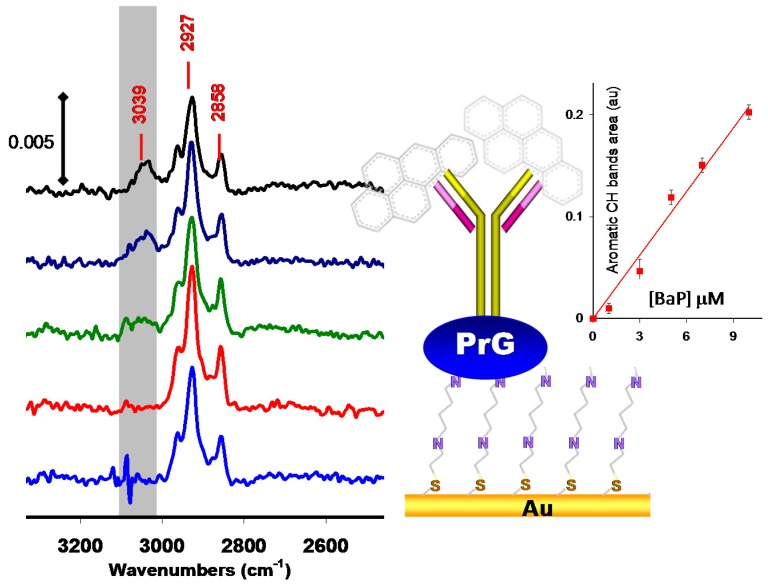
Direct detection of benzo-[a]-pyrene using PM-IRRAS immunosensors adapted from reference [43].

A very limited number of label-free biosensors have been set up to detect and quantify B[a]P due to very small size of this molecule (252.31 g·mol^−1^, All these biosensors operate in the competitive format with either electrochemical [40], optical [41], or piezoelectric [42] transduction modes. The unique reported example of direct biosensing of B[a]P was done using PM-IRRAS [43,44] (Figure 4). PMIRRAS made it possible to directly detect PAH compounds thanks to their specific ν_CH_ aromatic signals at ca ν = 3030 cm^−1^ with a limit of detection below 3 μM [22,43.^44^]. This particular case of B[a]P detection highlights the potential of surface IR in detecting very small targets but, more important, it shows the potentiality of this technique to provide an accurate way to discriminate specific and non-specific interactions by detecting the specific spectroscopic signature of the desired target.

### 2.4. Localized Surface-Plasmon Field-Enhanced IR Spectroscopy

A new chapter in surface-sensitive IR spectroscopies was opened with the introduction of various metallic nanostructure formats prepared on the IR substrate surface [45] aiming at adding localized surface-plasmon field enhancement mechanisms to further increase the optical field for improved sensitivity in monitoring interfacial bioaffinity reactions.

**Figure 5:**
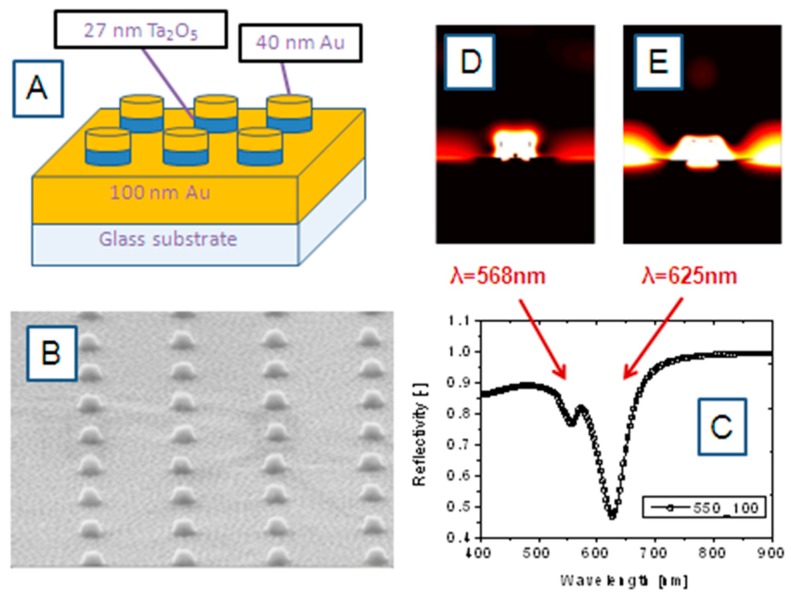
(**A**) Schematic arrangement of the Au nanodisc array for collective excitation of coupled propagating and localized surface plasmon modes; (**B**) SEM image of the nanodisc array; (**C**) reflectivity spectrum of a nanodisc array with a disc diameter of d = 100 nm, and a disc-disc separation distance of s = 550 nm. The (simulated) spatial distribution of the optical intensity associated with the two resonance modes at λ = 568 nm and λ = 625 nm, seen in the reflectivity spectrum are given in (**D**) (λ = 568 nm) and (**E**) (λ = 635 nm), respectively.

Surface plasmon resonance (SPR) spectroscopy in general has become widely used in the fields of chemistry and biochemistry for the characterization of surfaces and to monitor binding events of various analyte molecules. The use of plasmon surface polaritons as interfacial light has been discussed in the literature in great detail. In the classical case of propagating surface plasmon modes, the nearly free electron gas in a thin (~50 nm) noble metal film evaporated onto the base of a high-refractive index prism acts as an oscillator that can be driven by the electromagnetic wave impinging upon that interface. In the more recently introduced mode of operation, one deals with localized surface plasmon (LSP) resonances, in nanostructured noble metals, like arrays of nanoparticles, nanorods, nanodiscs, or nanoholes. Resulting again from the coherent oscillation of the conduction electrons in nanostructured noble metals upon the excitation by light one can achieve enormous optical field enhancements that can be directly translated into higher sensitivities in spectroscopic platforms. Figure 5 demonstrates the basics of one of the recently introduced formats [46], a combination of propagating and localized surface plasmon modes.

Figure 5A gives the schematics of the nanostructured surface architecture fabricated by focused ion beam patterning and lift-off structuring protocols. Onto the glass substrate a 100 nm thin Au layer, a rectangular array of 27 nm thin Ta_2_O_5_ nanodiscs was deposited. Each of the nanodisks is then covered with 40 nm thin Au. The disc-disc-separation distance was fixed at 550 nm. Figure 5B gives a SEM image of the IR substrate architecture. This pattern allows for the excitation of combinations (hybrid modes) of propagating as well as localized surface plasmon modes. An example for the reflectivity spectrum taken with such a nanodisc array with discs of d = 100 nm in diameter is given in Figure 5C. It shows two resonances, at λ = 568 nm and λ = 625 nm, with their local optical field distribution given in Figure 5D,E, respectively.

**Figure 6:**
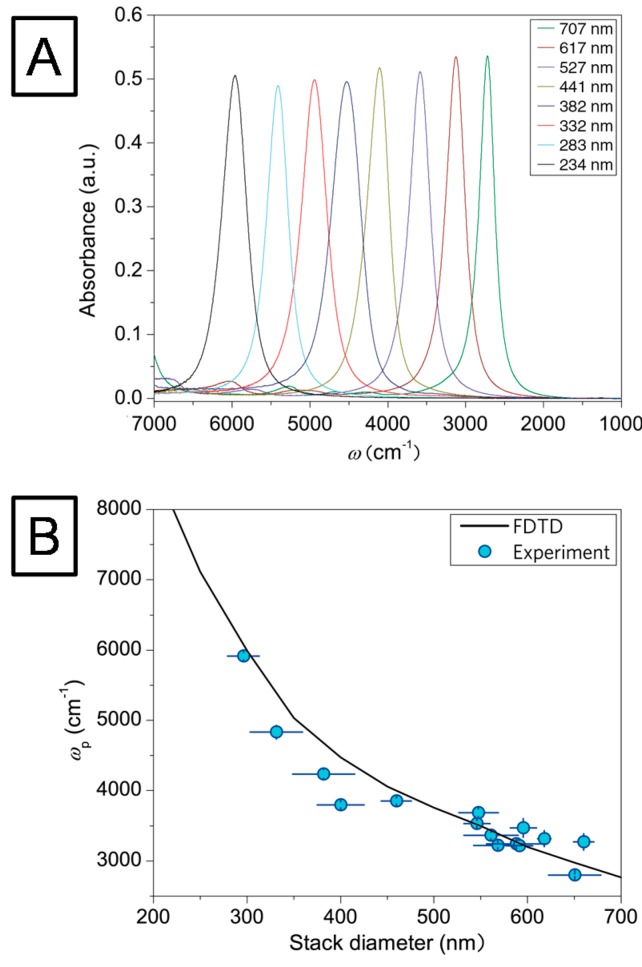
(**A**) IR absorption measurements of nanodisc arrays showing the tunability of the plasmon resonances by variation of the disc diameters; (**B**) Blue circles represent the measured spectral positions of the IR resonances of the nanodisc arrays. The black curve was calculated for a three dimensional model of the nanodisc array using finite difference time domain (FDTD) simulations, in good agreement with the experimental data.

In order to establish the relationship between the hybrid nanodisc diameter (at constant disc-disc separation s = 550 nm) and the resulting plasmon resonance frequency, infrared reflection-absorption measurements were performed on a dry-air purged infrared microscope (Bruker Hyperion 3000) coupled to a liquid nitrogen cooled mercury-cadmium telluride detector [46]. IR spectra of structures with different disc diameters were recorded in the spectral region between ν = 8000 cm^−1^ and ν = 600 cm^−1^, respectively, and calculated as co-addition of 300 scans. The obtained spectra were compared with a simulation model implemented in the MIT Electromagnetic Equation Propagation software package, which utilizes the finite difference time domain (FDTD) method [47]. Figure 6A shows the absorption spectra of the uncoated disk pattern for various disc diameters (250–700 nm). The strong absorption signals from the plasmons are in excellent agreement with the FDTD-simulations (*cf.* Figure 6B). The deviations between the measured resonance positions and the simulation are attributed to fabrication imperfections [46].

**Figure 7:**
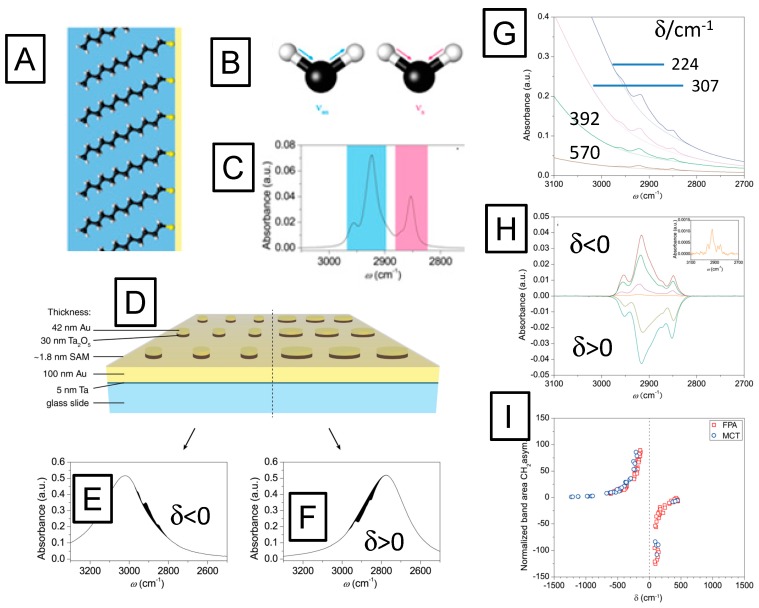
(**A**), schematic representation of a self-assembled monolayer of dodecanethiol on gold; the hydrocarbon chains consisting of CH_2_ groups and a CH_3_ terminal group; (**B**) schematics of the asymmetric (blue) and symmetric (magenta) stretching modes of CH_2_ groups: (**C**) IR spectrum of the asymmetric (blue) and symmetric (magenta) stretching modes of CH_2_ vibrations; (**B**) ATR IR absorption spectrum of dodecanethiol corresponding to the stretching modes depicted in (B); (**D**) Schematic picture of two-dimensional arrays of Ta_2_O_5_/Au nanodiscs with different radii, however, identical disc-disc separation distance; (**E**) reflection/absorption spectrum of a nanodisc array with the spectral position of the plasmon excitation at higher wavenumbers than the molecule vibrations (spectral difference δ < 0) enhances the molecular absorption, while the array with larger particle diameters (**F**), showing a plasmonic resonance at lower wavenumbers than the molecular vibration, *i.e.*, with spectral difference δ > 0, suppress the molecular vibrations which lowers the total absorbance; (**G**) infrared absorbance spectra of dodecanethiol monolayers adsorbed onto nanodisc arrays. The intensities of methyl and methylene stretching bands increase for lower δ values, *i.e.*, for structures which exhibit the plasmon resonance closer to the molecular vibrations; (**H**) baseline-corrected IR spectra of the C–H vibrations for different δ-values. Negative δ-values lead to enhanced absorption, whereas positive δ-values result in negative difference spectra; (**I**) normalized band areas measured at various spectral differences.

The potential of these hybrid plasmonic IR substrates for label-free biosensing applications by vibrational spectroscopies was tested by IR measurements of a dodecanethiol monolayer (*cf.* Figure 7A), self-assembled from solution onto Au nanodisc arrays. This model system offers well-described molecular vibrations, with the asymmetric and symmetric CH_2_ methylene-stretching vibrations (schematically given in Figure 7B) at ν = 2850 cm^−1^ and ν = 2925 cm^−1^, and the CH_3_-methyl symmetric and anti-symmetric stretching vibrations at ν = 2870 cm^−1^ (ν_s_) and ν = 2960 cm^−1^ (ν_as_), respectively, being the most prominent ones. The reference spectrum of dodecanethiol in solution was obtained by attenuated total reflection infrared (ATR-IR) measurements and is shown in Figure 7C. 

In order to test the influence of the plasmonic resonance of the nanostructured substrate on the IR spectra of the SAM, the diameters of the disks were systematically varied to tune the plasmon resonances of the SAM-coated substrate to the spectral range of the characteristic band positions of the CH stretching vibrations of the dodecanethiol molecules, *cf.* Figure 7D. The resulting IR spectra show that the spectral difference between the wavenumber of the molecular vibration, ν_m,_ and the wavenumber of the plasmonic resonance, ν_p_, *i.e.*, δ = ν_m_ − ν_p_ crucially influences the enhancement of the absorption signal. Figure 7 illustrates that the spectral difference δ determines whether the absorption of the molecular vibration is enhanced or suppressed. For negative values of δ, *i.e.*, for substrates with their plasmonic resonance being located at wavenumbers higher than the molecular vibrations, the characteristic absorption of the molecule is enhanced (Figure 7E). For positive values of δ, the effect reverses and the molecular absorbance is suppressed by the plasmon (Figure 7F), in analogy to strong damping in coupled oscillator modes for destructive interference [48,49].

The efficiency of the enhancement or the suppression depends on the spectral differences between both frequencies (*cf.* Figure 7G,H, respectively). The coupling between plasmon excitation and molecular vibrations already starts at extreme distances. While infrared spectra of the molecular monolayer could not be measured on a flat gold substrate (*i.e.*, with no plasmonic structures present), already nanodisc arrays with a plasmonic resonance at a spectral difference of δ = 1300 cm^−1^ relative to the molecule vibrations allowed for the detection of their characteristic absorption bands, as shown in the inset of Figure 7H.

In order to evaluate the coupling strengths for constructive and destructive interferences quantitatively, the band area from this weakly coupled case was used as a reference. Comparing the band area of the asymmetric stretching mode at ν = 2916 cm^−1^ for different plasmonic resonances to this reference revealed extremely strong growth in the band area. Reducing the distance from 1000 cm^−1^ to 80 cm^−1^ resulted in a signal enhancement of the molecular vibration by a factor of 125, both constructively as well as destructively (Figure 7I). It is important to stress that this factor is not an enhancement factor in the conventional sense, since it is normalized to an already enhanced absorbance signal. When scaled properly [50] the signal enhancement is found to be 2.2 × 10^6^ [46].

## 3. Surface Enhanced Raman Scattering and Biosensing

Surface Enhanced Raman Scattering (SERS) is a powerful tool to measure, identify and detect chemical or biological species. This technique has been widely used to develop highly sensitive sensors [51,52].

### 3.1. Principles of SERS

**Figure 8:**
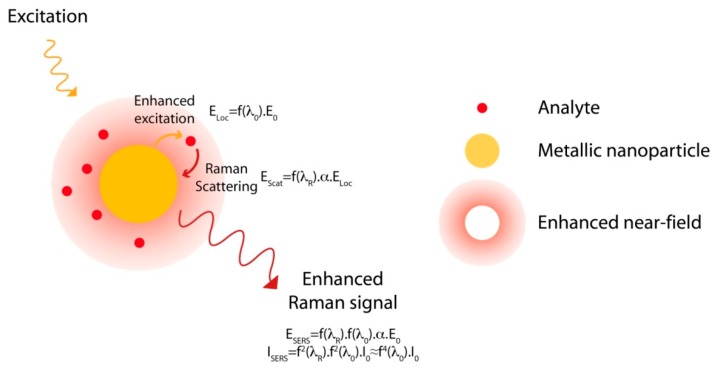
Principle of the Surface Enhanced Raman Scattering.

Raman scattering is a vibrational spectroscopy like IR. Similarly to IR spectra, Raman spectra are directly related to the molecular structure of the analyte, to its geometry and its conformation. The Raman spectrum of one molecule is a sort of fingerprint and this spectral signature allows its direct and specific identification. Raman can be seen as a good alternative to the fluorescent methods for small molecules biosensing. Unfortunately, this technique lacks sensitivity; Raman scattering is very weak since the Raman cross section, estimated to be between 10^−28^ and 10^−30^ cm^2^, is more than 10 orders of magnitude less than the fluorescence cross section, estimated to be around 10^−16^ cm^2^ [53,54]. Signal enhancement is therefore mandatory to use this technique for the detection of molecules at low concentration. In fact, the amplitude of the dipole moment (P) induced by the Raman scattering of the light is directly proportional to the amplitude of the electric field (E) used to excite the molecules through the relation P = αE, where α is the polarisability of the molecule. Thus, in normal conditions and without any enhancement, the Raman signal intensity is proportional to E^2^. As a consequence, any enhancement of the electric field induces an enhancement of the Raman scattering and of the Raman signal. This enhancement can be achieved by exploiting the optical properties of metallic nanostructures and more specifically by the excitation of localised surface plasmon (LSP). The strong enhancement of the electric field at the nanoparticle vicinity, induced by LSP, makes the molecules close to the surface scatter an en-hanced Raman signal. This effect, discovered in 1974 by Fleischmann *et al.*, [55] is called SERS (*cf.* Figure 8) [56,57]. As illustrated on the Figure 8, two different enhancement processes occur in SERS: an enhancement of the excitation light (enhancement of the electric field at the vicinity of the nanostructure surface due to the excitation of the plasmon resonance) and an enhancement of the Raman scattering (re-radiation process of the Raman signal). First the metallic nanoparticle is excited by the incident field, E_0_, and it produces an enhanced local field at the particle vicinity. This field can be expressed as E_Loc_ = f(λ_0_)∙E_0_, with f(λ_0_) the enhancement factor of the excitation field E_0_ at the excitation wavelength, λ_0_. In this case, any molecule located at the surface of the nanoparticle will be excited by this enhanced local field E_Loc_ and will scatter a Raman signal at a shifted wavelength, λ_R_. The amplitude of the Raman scattering, P, is then proportional to E_Loc_ as P = α∙E_Loc_= α∙f(λ_0_)∙E_0_. At this point, a second process, called re-radiation process, occurs. Indeed, as the Raman scattering is an electromagnetic field, it can also interact with the nanoparticle. This interaction may induce an enhancement of the Raman scattering calculated as:

E_Scat_ = f(λ_R_)∙P = f(λ_R_)∙f(λ_0_)∙α∙E_0_ = E_SERS_

with f(λ_R_) the enhancement factor of the Raman scattering at λ_R_. Finally, due to both enhancement processes, the Raman signal experiences two enhancement factors at the excitation and the Raman wavelengths, λ_0_ and λ_R_, respectively. The SERS intensity is then determined as I_SERS_ = f^2^(λ_R_)∙f^2^(λ_0_)∙I_0_ with I_0_ the Raman intensity without the nanoparticle (I_0_ =α^2^∙E_0_^2^). Since the Raman shift is low, both excitation and Raman wavelengths are close and both factors can be estimated to be nearly equal as f(λ_R_) ≈ f(λ_0_). Thus, the SERS intensity can be approximated as I_SERS_ ≈ f^4^(λ_0_)∙I_0_. Thus it is estimated that the Raman enhancement can be approximated as the power fourth of the enhancement of the electric field near the surface [58]. The Raman enhancement can then be huge since even a low field enhancement can induce a large Raman enhancement. Enhancement factor as high as 10 orders of magnitude has been measured [59] and single molecule detection has been reported [60,61].

This effect is highly local since it is estimated that the decay length of the LSP is in the order of a few tens of nanometers. As an example, for individual cylindrical particles (diameter 100 nm and height of 30 nm) produced by metal evaporation and for individual triangular nanostructures (edge 100 nm and height 30 nm) produced by nanosphere lithography, LSP decay lengths between 15 and 20 nm have been reported [62,63,64]. It has also been shown that this decay length depends strongly on the nanostructure size and geometry. Thus in the case of nanotriangles with width from 70 to 100 nm and height from 30 to 35 nm, the decay length increases when the nanotriangles width increases whereas it decreases when the height increases. However, since the Raman signal in SERS can be approximated to be proportional to the fourth power of the enhancement of the electromagnetic field amplitude around the nanoparticle, the Raman enhancement length is even more confined than the field decay length. Thus, Dieringer *et al.* have shown that the distance dependence of the SERS intensity, I_SERS_, can be approximated as:

I_SERS_ = (1 + r/a)^−10^

with r, the distance from the nanoparticle surface and a, the average size of the field enhancement from the surface [64]. They notably demonstrated that in the case of Ag films deposited on latex beads (AgFON surface), a = 12 nm and that the I_SERS_ is divided by ten at a distance of 2.8 nm. The SERS intensity decreases very fastly and is nearly null for distance of a few nanometers from the nanostructure surface.

Finally, it has been demonstrated that the Raman enhancement is strongly related to the position of the LSP resonance and that it can be optimised and controlled precisely by fine tuning of the geometrical parameters and the optical properties of the nanostructures [58]. SERS can then be used to develop highly sensitive and reproducible sensor.

### 3.2. Sensors Based on SERS

SERS-based sensors have several advantages. First, they are label free since the Raman spectrum is a spectral signature of the analytes. Second, they are highly sensitive due to the high enhancement provided by the SERS and some concentrations lower than the femto-mole can be detected. Third, the Raman bands are thin compared to the fluorescence bands, which allows observing and distinguishing several analytes on the same Raman spectrum and paves the way to the multiplexing detection.

**Figure 9:**
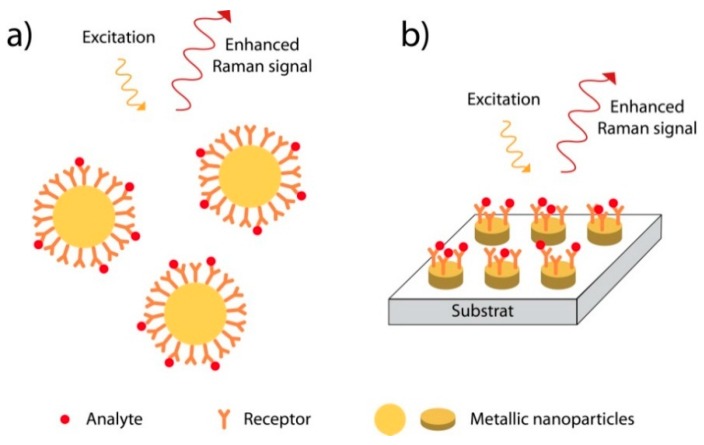
Principle of SERS sensors (**a**) in solution or (**b**) on surface.

To develop SERS sensors, different types of nanostructures were used: nanoparticles either dispersed in solution or deposited on a surface, and nanostructured metallic surfaces (Figure 9) [51,58]. In the first case, the colloidal nanoparticles are easy to produce by chemical methods but the size distribution as well as the aggregation of the nanoparticles have to be finely controlled to provide a reproducible SERS signal. In the second case, the nanoparticles are produced by physical methods such as lithography techniques (nanosphere lithography, electron beam lithography, nanoimprint…). These methods allow a precise control of the geometrical parameters of the nanostructures at the nanometer scale but are expensive and time consuming.

### 3.3. Detection of Chemical Compounds

Several SERS-based sensors have been described for small molecules detection. Self-assembled monolayers or polymer layers have been used to functionalize the nanoparticle surface for the subsequent immobilization of a specific molecular receptor. In most of the cases, receptor-analyte interactions are non-covalent and hydrophobic ones. For polar molecules, these interactions can also be electrostatic.

For example, pentachlorophenol (PCP) has been detected in the range 0.5 to 100 μM using silver nanoparticle aggregates decorated with cysteamine SAM [65]. The cysteamine has provided both rapid target molecule adsorption and internal reference of a quantitative sensing. The detection of chlorobenzene and phenylacetylene (limit of detection close to 100 and 10 ppb, respectively) has also been demonstrated with gold and silver nanoparticles encapsulated in a tetraethoxysilane (TEOS) and methyltriethoxysilane (MTEOS) layers [66]. The authors have shown that the silver nanoparticles with the MTEOS layer provide a higher detection limit than TEOS. Another approach is to graft the gold nanoparticles directly at the surface of polystyrene beads deposited on silanized quartz substrates [67]. The pre-concentration of naphthalene via hydrophobic interactions has been achieved at the beads surface and allowed its detection in the range 1 to 20 ppm (7.8–160 μM) in aqueous media. Finally, the sensor selectivity can be improved by employing molecularly imprinted polymers (MIPs). This approach has been exploited to detect the 2,4,6-trinitrotoluene (TNT), an explosive agent. A detection limit in the pM range has notably been demonstrated by Yang *et al.* [68].

Another reported strategy is based on the use of host-guest interactions with molecules such as cyclodextrines (CD) or calixarenes (CX). CD deposited on a rough silver surface were successfully used to detect several pollutants such as benzene, toluene, *m*-xylene, chlorobenzene, and *m*-dichlorobenzene, with detection limits of 10,000, 400, 1400, 250, and 15 ppm, respectively [69]. Xie *et al.* have described quantitative detection of anthracene, pyrene, chrysene, and triphenylene with a β-CD grafted on gold and silver nanoparticles with limits of detection of 100, 10, 100, and 1000 nM, respectively [70,71]. The same method has also been utilized to detect 2,4-dinitrotoluene (DNT) at a concentration of 10 pM [72] and methyl parathion insecticide at 1 pM [73]. For CX, polycyclic aromatic hydrocarbons such as benzo[c]phenanthrene, triphenylene, and coronene have been detected in concentration range between 10 and 100 ppb [74]. Using the appropriate surface functionalization, one can notice that it is possible to develop some highly sensitive and selective SERS sensor with limit of detection in the range of the nM or the pM.

### 3.4. Detection of Biomolecules

Even if the biological molecules have complex Raman spectrum with a large number of bands, they all exhibit some specific features and it is possible to define spectral signatures using the Raman spectrum. This spectroscopic method is then intensively used to identify the biomolecules [75] and to observe their structure and their conformational changes through biological mechanisms [76,77,78].

In this context, the detection of proteins [79] or of DNA [80,81] directly deposited on metallic nanoparticles has been demonstrated. In the case of proteins, it has been shown that the SERS spectra are different from the ones recorded by classical Raman, either in solution or in solid state. This can be explained by the change in protein conformation due to the interaction with the metallic surface [79]. Such changes have also been observed at different temperatures [82]. A statistical analysis (principal component analysis, PCA) of the different observed spectra can allow the determination of specific conformation or orientation of the biomolecules at the metallic surface as demonstrated by Brulé *et al.* [83].

Such statistical analyses are also essential when physiological fluids are studied. Indeed, since all the molecules that are deposited at the surface of the metal will exhibit an enhanced Raman signal, the SERS spectrum will correspond to the sum of the contribution of all the components of the fluid. The analysis of the spectrum is then necessary to highlight some spectral changes due to the modifications of the fluid composition in the case of a disease (over-expression of a specific biomarker or changes in the relative concentrations of the fluid compounds). For example, Premasiri *et al.* have observed the release of the hypoxanthine in the blood after several hours of storage [84]. All the same, Lin *et al.* have used the PCA method to differentiate the blood serum taken from colorectal cancer patients [85].

Using silver film under nanosphere substrates covered by a dodecanethiol/mercaptohexanol SAM layer, the *in vivo* detection of glucose has been demonstrated in an animal model and it was possible to measure the glucose variation on a concentration range between 10^−3^ and 300 × 10^−3^ mol/L [86,87]. By using the same SERS substrate, the detection of *Bacillus* spores with a detection limit of 10^−14^ mol/L has also been demonstrated [88]. Recently, SERS sensors based on the use of aptamers have been proposed. First, the selective detection of the ochratoxin (OTA) has been proposed using triangular nanoparticles produced by nanosphere lithography and functionalized with an aptamer [89]. Second, the indirect detection of melanine has been shown by Wen *et al.* [90]. Silver nanoparticles with a diameter of 10 nm modified by oligonucleotides were used as SERS substrates. The detection was done through the observation of the nanoparticles release induced by their interaction with the melanine. This process has been monitored by SERS since the nanoparticles aggregated and produced hot spots. With this system melamine concentrations ranging from 6.3 to 403.6 μg/L have been measured.

## 4. Tip Enhanced Raman Scattering

The concept of the SERS can also be applied to a nanometric metallic tip such as scanning microscope tip. This technique is known as Tip Enhanced Raman Scattering. The tip is then used to enhance locally the electric field and it acts as an enhanced light source at the nanometer scale. This light source can be used to excite locally a material and probes its properties. By scanning the material surface with the tip, it is possible to make a Raman imaging. Since the nano-source produced by the tip is highly confined and localized at the tip apex, the observation area is largely reduced. The confinement is essentially given by the size of the tip apex radius commonly comprised between 10 and 50 nm. The optical resolution of the imaging, meaning the ability of the set-up to resolve spatial information from a specific area, can then be reduced considerably down to the 10 nm scale [91], or even lower since several groups have reached nanometer or subnanometer resolution [92,93]. As an example, Chen *et al.* have demonstrated 1.7 nm resolution on the chemical analysis of carbon nanotubes [92] or Zhang *et al.* have been able to image the inner structure of the TBPP molecules with subnanometric resolution [93]. Thus, TERS allows a complete characterization of the chemical composition of surfaces and interfaces and can provide a physical/chemical imaging with high spatial resolution and enable the observation of a very small quantity of sample (probe volume of few tens of nm^3^ or observation of individual molecule). TERS can be seen as an ultra-sensitive and label free characterization technique.

TERS has already demonstrated its performances in the characterization of several materials as carbon nanotube [94,95,96,97], graphene [98,99,100], semiconductors layers (InN [101], Si(001) [102], GaAs(001) [103]), oxides layers (BaTiO_3_ [104], TiO_2_ [105]) or molecular monolayers (copper porphyrin [106], copper phthalocyanine (CuPc) [107], thiol compounds [108,109]), and biomolecules (proteins [110], peptides [111,112], single RNA strands [113], cytochrome [114], lipid domains [115], alginate biofilms [116], insulin amyloid fibrils [117,118,119]).

However, this technique not only gives information on the structures of molecules or crystals. It can also be used to monitor some structures changes and some chemical reactions. For example, it enables some stress measurements [120] or the imaging of the strain distribution in carbon nanotubes [121]. Using TERS, it has also been possible to monitor some catalytic reactions and processes [122,123,124] as well as some biological processes (proteins interactions [125] or protein-ligand binding [126]).

To improve the TERS sensitivity, some proposed to deposit the molecules to be characterized on a metallic surface used as substrate. In this case, the coupling between the metallic tip and the metallic surface create a resonant optical cavity with the excitation of a Gap Mode plasmon. Large field enhancement occurs that leads to a strong enhancement of the TERS signal and enhancement factor up to 10^7^ has been measured [127,128] Using such configuration and low temperature ultra-high vacuum conditions, it was possible to achieve submolecular spatial resolution on a single TBPP molecule deposited on Ag surface [93].

This enhanced spectroscopy can be also applied to the IR range. This technique allowed imaging in the mid-infrared region with resolution of few tens of nanometer [129,130] This method can be used to characterize solid state materials (Si0 [131], Si3N4 [132], strains in SiC [133], polymers [134]), as well as nano-objects [135,136] or bio-objects [137]. 

## 5. Conclusions

Biosensors are analytical devices incorporating a biological material (a receptor) intimately linked to a physicochemical transducer. These devices are designed with the objective of detecting a target, specifically and rapidly, even at trace amounts and even in a complex environment. One of the greatest challenges in the field of biosensors is their sensitivity. This is particularly true in the case of small-sized target analyte sensing (biomarkers, haptens, toxins, odorants, *etc.*), as the response of classical detection techniques is generally below the required detection limit. 

The detection sensitivity of biosensors can be increased either by enhancing the performance of the biological material or by amplifying the signal measured by the transduction techniques to be used. Substantial progress has been made recently in increasing the avidity/affinity of bioreceptors, being either antibodies, aptamers, or specific “selectors”, designed to recognize and bind small-sized targets from solution or from air. 

On the other hand, SERS and SEIRAS that provide the identification of the analytes as well as any structural modifications due to the interaction with the bioreceptor, through the spectral signature recorded by these vibrational spectroscopies have also seen significant progress. The signal used to identify the targeted analytes is their vibrational spectra measured by IR or Raman spectroscopy. Such signal is directly related to the molecular structure and can be seen as a direct signature of the analyte. Recent years have seen an enormous gain in sensitivity by smart modifications of the experimental design of both families of sensing platforms. As we have summarized in this contribution, such enhanced spectroscopies exploit the plasmonic properties of specific metallic substrates that create a highly intense electromagnetic field at the vicinity of the interface to the analyte solution. This enhanced electromagnetic field will induce an enhancement of the Raman scattering and of the IR absorption. The enhancement factors in SERS and SEIRAS have been estimated to be close to 1010 and 106, respectively, and have allowed for the observation and the detection of very small amounts of molecules paving notably the way for the single molecule detection. 

Metallic NPs, including metallic nano-tips of scanning microscopies and spectroscopies, have opened yet another dimension of vibrational spectroscopies for small molecule biosensing. They have specific optical properties depending on their chemical nature, size or on their geometry. In the visible range, one can excite collective oscillation of the free electron gas confined to the three dimensions of the nanoparticle or the tip, known as localized surface plasmon (LSP). In this situation, the LSP resonance condition depends on the metallic nature (effect of the metal permittivity) and on the geometry (effect of the confinement of the electron cloud) of the nanostructure. This LSP resonance can then be tuned on a spectral range from the visible up to the near IR in the case of gold or silver nanostructures. The excitation of the LSP induces a large enhancement of the electromagnetic field at the nanoparticle surface vicinity. Such local field enhancement can be exploited to further enhance the vibrational fingerprints of molecular groups in the proximity of a metallic NP. As a consequence, specific optical properties as well as the Raman signal enhancement can be controlled and tailored by the fine control of the NA geometry.

We have demonstrated that we are able to define some specific rules to achieve the optimal enhancement in SERS and SEIRAS depending on the plasmonic nanoparticle parameters: size and shape, metal nature, plasmon position, nanoparticle configuration, the incident light polarization, *etc*. We have exploited these rules to design plasmonic nanostructures in order to achieve the highest enhancement factor and thus to reach the highest sensitivity as well as the lowest detection limits. 

It is expected that these enhancement levels will prove to be useful also for the commercial exploitation of vibrational spectroscopies for the detection of small analyte molecules in (bio-) medical application, for bioprocess control, for food quality or environmental monitoring, or in crop disease detection.
